# Nanoparticle assembly enabled by EHD-printed monolayers

**DOI:** 10.1038/micronano.2017.54

**Published:** 2017-09-11

**Authors:** Benjamin Francis Porter, Nhlakanipho Mkhize, Harish Bhaskaran

**Affiliations:** 1 Department of Materials, University of Oxford, Oxford OX1 3PH, UK

**Keywords:** additive nanomanufacturing, dual-harmonic kelvin probe microscopy, electrohydrodynamic printing, self-assembly, 24 nanoparticles

## Abstract

Augmenting existing devices and structures at the nanoscale with unique functionalities is an exciting prospect. So is the ability to eventually enable at the nanoscale, a version of rapid prototyping via additive nanomanufacturing. Achieving this requires a step-up in manufacturing for industrial use of these devices through fast, inexpensive prototyping with nanoscale precision. In this paper, we combine two very promising techniques—self-assembly and printing—to achieve additively nanomanufactured structures. We start by showing that monolayers can drive the assembly of nanoparticles into pre-defined patterns with single-particle resolution; then crucially we demonstrate for the first time that molecular monolayers can be printed using electrohydrodynamic (EHD)-jet printing. The functionality and resolution of such printed monolayers then drives the self-assembly of nanoparticles, demonstrating the integration of EHD with self-assembly. This shows that such process combinations can lead towards more integrated process flows in nanomanufacturing. Furthermore, in-process metrology is a key requirement for any large-scale nanomanufacturing, and we show that Dual-Harmonic Kelvin Probe Microscopy provides a robust metrology technique to characterising these patterned structures through the convolution of geometrical and environmental constraints. These represent a first step toward combining different additive nanomanufacturing techniques and metrology techniques that could in future provide additively nanomanufactured devices and structures.

## Introduction

Developing new, complex types of nanotechnology for markets is constrained by the workflow of modern nanomanufacturing, with entire assembly runs being inflexible from beginning to end. Thus, although existing nanomanufacturing techniques are extremely effective at large scale manufacturing with low per-unit costs, they still incur the traditional limitations of manufacturing, which is that high volumes are necessary to justify retooling the process. Additive manufacturing such as three-dimensional (3D) printing technologies have served to fill this gap for lower volume manufacturing of late, but such techniques have not made it into the nanoscale domain. Burgeoning methods of additive nanomanufacturing offer a potential solution to this problem, by enabling the top-down integration of existing devices with nanomaterials^1–6^, whose properties have demonstrable applications in novel areas of biosensing^4^, single-electron transport^5^, quantum phenomena^6^, and more. These materials can be made to self-assemble into desired configurations via many driving forces, from electrophoresis^7–10^, DNA-linkages^11,12^ and geometrical interactions^13–15^. Controlling when and where such self-assembly occurs requires lithography, however, which to integrate with modern device designs must meet sub-100 nm resolution as standard. Of the top-down methods in development today, electrohydrodynamic (EHD) jet printing is the most promising option to achieve fabrication to this scale, and is already established as a technique capable of sub-50 nm deposition of conductive materials^16,17^, including 3D structures^17,18^, and larger functional structures^19^. Combining such methods of nanoscale printing with self-assembly would open-up a pathway for inventors to integrate nanoparticles and their unique properties into established industrial manufacturing processes.

Although EHD has several key advantages, such as its ultrahigh-resolution capability with high diameter tips^16,17^, 3D printing capability^17,18^ as well as the ability to pattern without high vacuum requirements, there have been few instances where EHD has been successfully combined with other additive techniques, that is, self-assembly. A key advance that is required in additive nanomanufacturing is the ability to combine different nanoscale techniques, approaching 10 s of nm. This frequently requires patterning of materials that are not very conductive such as molecular monolayers, followed by self-assembly techniques—something not demonstrated for EHD printing thus far. Furthermore, combining assembly with high quality metrology at the nanoscale is also a challenge that must be addressed^20^; at the macroscale, visual metrology can be easily used to make accurate judgements on intermediate steps, which is challenging at the nanoscale. So any manufacturing process development will need to be developed alongside complementary metrology tools at an early stage, as such tools are not merely for quality control, but in most cases are for determining whether or not a process has even succeeded.

In this paper, high-resolution EHD printing is combined with self-assembly techniques to demonstrate that such blends of different techniques can produce a rapid yet adaptable nanomanufacturing process in an additive manner (i.e., without lithography). To achieve this, we started by optimizing the electrostatic assembly process defined using electron-beam lithography, to drive electrostatically-stabilized nanoparticles onto positively charged templates. We also investigated the benefits of exploiting geometrical features with the process—this is important to understand how processes vary with geometrical variations, which would be present if integrated with pre-existing devices. We then examine an additive method for patterning these devices through EHD printing of functional amino-silane compounds, demonstrating for the first time that it is also capable of printing molecular monolayers onto surfaces down to hundreds of nanometers, and prove their post-printing functionality through further electrostatic nanoparticle assembly. Finally, we demonstrate that the mid-process metrology of these surfaces is possible through Dual-Harmonic Kelvin Probe Microscopy (KPM)^21^, showing this can accurately define the metrology of features even under active liquid conditions—an emerging technique not commonly employed for such process metrology.

## Materials and methods

### Fabrication of electrostatic assembly devices

The initial self-assembly tests were performed on 10×10 mm chips with a 100 nm SiO_2_ surface with monolayers patterned using an electron-beam lithography (EBL) process. For Au-MHA devices, based on the previously established routes to single-particle assembly^7,8^, 50 nm of Au (with Cr adhesion) was thermally evaporated onto the surface under high vacuum. These surfaces were spin-coated at 4000 rpm with poly(methyl methacrylate) (PMMA) to a 100 nm thickness, which was then patterned by EBL. After development, the exposed metal areas were chemically etched down to the SiO_2_ surface using standard etching solutions (Sigma-Aldrich Co. 651826 & 651818). Following removal of the remaining resist, the chips were immersed in a 5 mM solution of (3-aminopropyl)triethoxysilane (APTES) in toluene for one hour, allowing a molecular monolayer of APTES to grow on the silica surface. This was followed by rinsing and immersion in a 5 mM solution of 16-mercaptohexadecanoic acid (MHA) in ethanol for 12 h allowing the MHA monolayer to form on the Au, followed by rinsing and drying with N_2_ gas.

Devices used for further self-assembly tests had the Au-MHA surface substituted for the electrostatic repulsion of the SiO_2_ surface, to establish that the assembled particles could potentially be integrated with silicon circuitry. PMMA was spin-coated directly onto the SiO_2_ chips and patterned by EBL. The chips were then immersed in 5 mM APTES in propan-2-ol (IPA) for 1 h, to allow the APTES monolayer to grow on the exposed silica surface without compromising the PMMA mask. The use of IPA in place of toluene as the solvent was necessary to prevent the dissolution of the PMMA during monolayer assembly. For recessed templates, the PMMA was preserved for subsequent nanoparticle assembly and for planar templates the PMMA was removed using propan-2-one.

### EHD printing of monolayers

Surfaces for printing were prepared on 100 nm thick, thermally grown SiO_2_. These surfaces were prepared for bonding with the monolayer by pre-cleaning in piranha solution (H_2_SO_4_:H_2_O_2_, 3:1). This was performed at 120 °C for 30 min to allow for full hydroxylation of the silica surface, followed by 10 min rinsing in hot deionised water and drying with a stream of N_2_ gas. These chips were then placed on a conductive ITO ground plate for printing to be performed. The inks were prepared by sonication for 15 min of APTES to allow full dissolution into the given solvent of either water, ethanol, IPA or triethylene glycol monomethyl ether (TGME). EHD printing nozzles were prepared by sputter deposition of ITO (75 nm thick) onto pre-pulled glass pipettes with an inner diameter of 1 μm (World Precision Instruments, Hitchen, UK). Nozzles were then immersed in a 5 mM solution in toluene of 1*H*,1*H*,2*H*,2*H*-Perfluorooctyltriethoxysilane (Sigma-Aldrich, Gillingham, UK) to make the oxide surface of the ITO hydrophobic. The ink for printing was injected into the pipette using micro-loading pipettes (Eppendorf, Stevenage, UK). The loaded capillary was then lowered to a 30 to 100 μm distance from the printing surface observed via a microscope. A voltage of 2 to 10 kV was applied between the capillary and the ground plate using a DC–DC converter and a DC power supply.

### Electrostatic self-assembly of nanoparticles

Nanoparticles were assembled onto devices from a 100 μL droplet of 20 nm, citrate-stabilized Au colloid, which was deposited onto the templated surface of the test chip. This droplet was left in place for a given time before being rinsed sequentially in deionised water and IPA and dried with a stream of N_2_ gas. For assembly times exceeding one hour, the chips were placed in a high humidity environment to prevent evaporation of the colloid.

### Sample characterization

Samples were characterized following printing of monolayers and assembly of nanoparticles using a combination of Atomic Force Microscopy (AFM), KPM, and Scanning Electron Microscopy (SEM). AFM and KPM scans were performed to characterize the morphology of EHD-printed monolayers on the SiO_2_ surface, using an Asylum MFP-3D AFM with 81.2 kHz silicon PPP-FMR Nanosensors AFM probes. Scanning Electron Microscopy was used for the characterization of nanoparticle assembly processes, imaging the particles after they were deposited on the features, and was performed with a Jeol 6500F SEM at 10 kV.

To enable the imaging of the features within the electrolyte, we used an offshoot of the KPM technique, Dual-Harmonic KPM (DHKPM)^22,23^. In this technique, the closed-loop feedback using the DC offset of the signal in KPM is replaced with a comparison between the different frequency components of the cantilever response during open-loop, electrostatic actuation of the cantilever. This leads to a new expression of surface potential in terms of the amplitude response, *A*, at the actuation frequency *ω* and 2*ω*:

(1)|VS|=14AωA2ωGωG2ω|VAC|

Here *G*_*ω*_ and *G*_2*ω*_ are the transfer functions for the cantilever when it is oscillating at *ω* and 2*ω*, respectively, which allow for us to convert the amplitude to the force acting on the cantilever at these frequencies. For instance^22^:

(2)Gω=1k1−(ωω0)2+(ωQω0)2

is the transfer function for the force at drive frequency *ω* for a cantilever with a spring constant *k*, a natural frequency of *ω*_0_ and a quality factor of *Q* under thermal excitation. Experimentally, DHKPM measurements were performed using 325 kHz HQ:NSC15/Cr-Au MikroMasch AFM probes, which were better suited to operating in liquid.

## Results

### Single-nanoparticle resolution via conventional lithography

**Figure 1:**
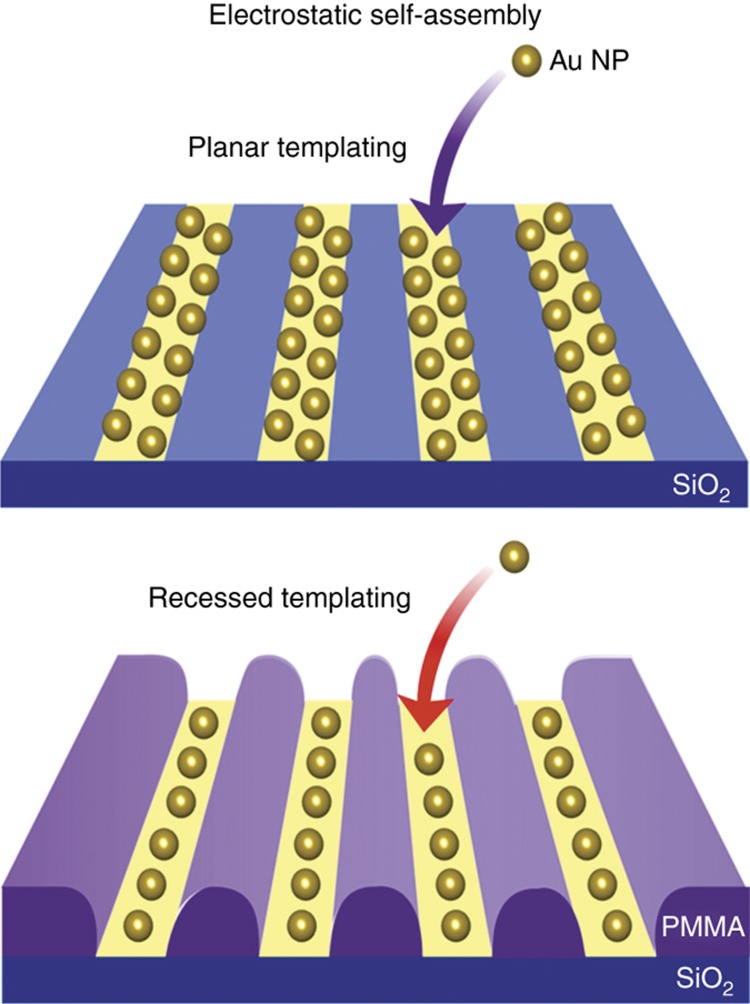
Planar and templated electrostatic self-assembly. Schematics showing the difference between planar and templated electrostatic assembly. In both cases, a charged nanoparticle approaches a surface that is patterned with electrostatically attractive (yellow) and repulsive (blue/purple) surfaces. The particles assemble onto the attractive surfaces and are repelled by the repulsive ones, which given sufficient time allows dense coatings of these nanoparticles to form on the surface. However, if the surface is templated so that the attractive surface is recessed relative to the repulsive surface then the funnelling from the repulsive surface becomes stronger. This reduces the width of the area coated with nanoparticles following assembly, which can push the resolution of assembly beyond that of the defined template. In the case of a template such as poly(methyl methacrylate) (PMMA), the surrounding area can be removed following assembly without affecting the functionality of the nanoparticles.

The concept for electrostatic assembly is presented in Figure 1. Many colloids of nanoparticles are stabilized by the mutual electrostatic repulsion between adjacent nanoparticles. If a surface is patterned with attractive and repulsive surfaces then nanoparticles will be electrostatically guided towards the attractive surface, whilst being repelled from the repulsive areas. When brought into contact with the nanoparticle colloid, the particles can thus assemble onto the attractive surfaces following the resulting electric field formed by the charges on the surface. Once an area is coated with nanoparticles, the mutual repulsion between particles inhibits further assembly, so the deposition forms a single-nanoparticle coating on the surface. The exact geometry of the surface can affect this process, for example, if the attractive surface is recessed within the repulsive one, then greater funnelling of the nanoparticles is expected to occur^24^.

**Figure 2:**
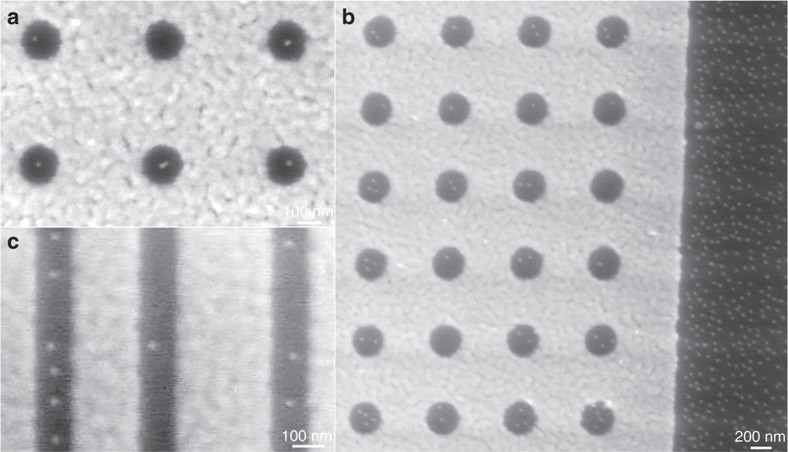
Electrostatic assembly with Au templates. Scanning electron microscopic (SEM) images of 20 nm Au particles (2×10^11^ per mL) electrostatically assembled from a colloidal droplet onto a APTES/MHA templated surface over 12 h. The surface is patterned with Au with the 16-mercaptohexadecanoic acid (MHA) monolayer bound to it (light) and the surrounding SiO_2_ surface is coated with a monolayer of APTES; the MHA and APTES monolayers are electrostatically repulsive and attractive to the nanoparticles respectively. In (**a**), single nanoparticles have been isolated into some of the circular regions ~130 nm in diameter. (**b**) A similar array of 250 nm diameter holes that contain a diverse number of particles. (**c**) Some particles funnelled into a 100 nm wide channel with single-particle width, though not consistently.

Initially, we looked at templated assembly on SiO_2_ surfaces patterned with Au, with a 16-mercaptohexadecanoic acid (MHA) monolayer bound to it (light) and the surrounding surface coated with a monolayer of 3-aminopropyl triethoxysilane (APTES) (dark); the MHA and APTES monolayers are electrostatically repulsive and attractive with respect to the negatively charged, citrate stabilized 20 nm Au particles. This design and assembly process is based on devices that have previously been shown to successfully assemble particles with nanoscale precision^7,8^. This process showed good results, with particles funnelled from the MHA to the APTES surfaces, with features ranging from 100 to 130 nm wide achieving capture of individual particles, showing great exclusivity (Figure 2). The low concentration of particles used in this process appears to have been a detriment to the efficiency of the process, however, with other similar processes achieving similar resolution over much shorter time scales. The Au-MHA template used in this process also inhibits the use of the particles for applications, as the Au-MHA obstructs the integration with other electrical devices and its removal would cause damage to the Au assembly.

**Figure 3:**
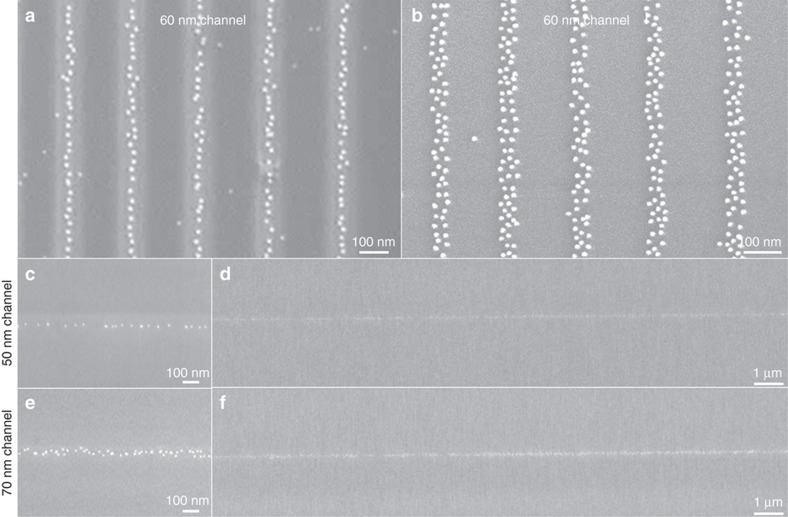
Superior electrostatic assembly with templated surfaces. 20 nm particles are assembled over 8 min from a colloidal droplet (7×10^11^ per mL) onto a surface of patterned electrostatically attractive APTES and a surrounding repulsive surface. In the case of a recessed surface patterned into 100 nm thick poly(methyl methacrylate) (PMMA) (**a**), the 20 nm particles are electrostatically funnelled to the centre of the channel to form lines one particle in width. In contrast, a planar surface (**b**) where the PMMA has been removed to leave hydroxylated, the nanoparticles undergo reduced funnelling to produce a coating approximately two particles wide. The PMMA can also be removed after assembly of the particles (**c**–**f**), which demonstrates that the funnelling of the recessed template can be preserved. The assembly is highly sensitive to minimal changes in topography, as evidenced by the results with (**c**) 50 nm and (**e**) 70 nm channels, but is shown to occur over both these nano and macroscales, with assembly occurring over any distance across the surface (**d** and **f**).

However, a simplified technique for single-particle resolution would be very useful for integration of further components. We, therefore, choose to focus on the direct patterning of APTES onto SiO_2_ surfaces without the Au-MHA template, instead utilizing the negative surface charge of hydroxyl groups in a coating of poly (methyl methacrylate) patterned on the surface. The chains of particles, one particle in width, such as for the 60 nm channels in Figure 3a, are commensurate with those observed for the Au-MHA template in Figure 2c except this time particle spacing is much smaller and the density of particles deposited is more consistent. The principal difference in the result arises from the higher concentration of particles in the colloid (7×10^11^ per mL) used in Figure 3a, being greater than the 2×10^11^ per mL used in Figure 2c. The higher particle concentration allows the assembly to occur more rapidly, with assembly requiring minutes rather than hours, and the spacing between particles to be significantly reduced. The reduction in particle spacing can in part be attributed to the reduction in ionic strength of the colloidal medium, which was lower for the Au-MHA template tests. Both the PMMA and Au-MHA templates were recessed into the surface, with the PMMA 100 nm in thickness in principle offering greater funnelling than the 50 nm thick Au-MHA surface. This would appear to indicate that the concentration of the colloid is the principal factor for determining interparticle spacing. With the greater particle density, a narrower channel was required to achieve funnelling to one particle width.

For other processes, it might be more efficient to be able to work with a planar surface. Removing the PMMA template produced patterned regions of APTES on the surface surrounded by hydroxylated silica, effectively flush with the surface save for the molecular thickness of the APTES. In Figure 3b, this is shown to achieve a wider coating of particles in a 60 nm channel of APTES, approximately two particles in width. This demonstrates that the funnelling of particles is increased when the surface is recessed rather than planar. Further to this, the PMMA template can be removed after assembly of the particle instead via standard cleaning with propan-2-one and is shown to have no detrimental effect on the particles deposited on the attractive SiO_2_-APTES surface, whilst removing those that had adhered to the PMMA surface (Figures 3c–f). This can result in single-particle-wide nanoparticle chains, which we show are consistently formed over long distances on the surface (Figures 3d and f). This process is very sensitive to small changes in the metrology of the template, with the width of chains formed on the surface varying from relatively inconsistent single-particle-wide chains in 50 nm channels (Figure 3c) to 2-particle-wide chains at 70 nm (Figure 3e).

### EHD printing of molecular monolayers

**Figure 4:**
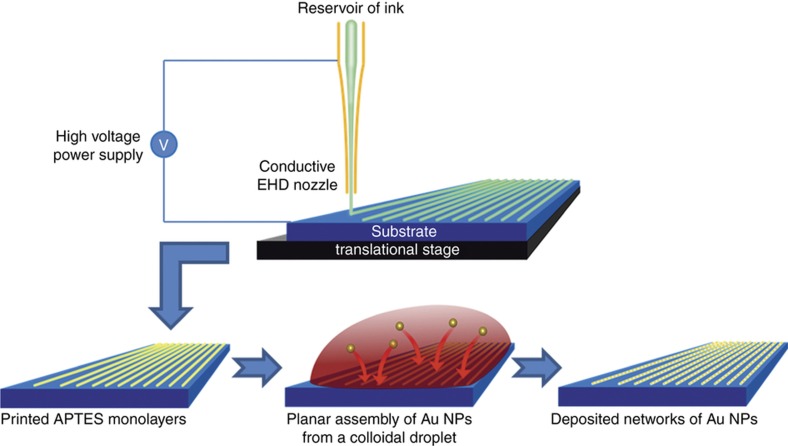
Process for electrohydrodynamic (EHD) printing of functional monolayers. In EHD printing, a capillary with a conductive coating is held at a high positive voltage above the grounded conductive substrate, whilst it is translated on the stage. Being able to control the exact motion of the tip is crucial in being able to achieve intricate patterning which would be difficult with other techniques. In our work, inks with dissolved APTES are printed to directly pattern functional monolayers on the substrate. These monolayers are then used to drive the self-assembly of 20 nm Au particles from a colloid to create precisely defined networks of Au nanoparticles (NPs).

Thus far, we have demonstrated that assembly of nanoparticles can be achieved rapidly onto templates defined using electron-beam lithography. Direct printing of these molecular templates is, however, the preferable option due to the flexibility this affords, which is necessary to develop additive nanomanufacturing techniques. Electrohydrodynamic (EHD) jet printing has re-emerged in recent years as an effective method of additive nanomanufacturing^25,26^, with the high-resolution patterning of electrode structures down to tens of nanometres already demonstrated^16,17^. The basic operation of the technique involves the printing of an ink onto a substrate from a small capillary nozzle by the application of a high potential difference. The resulting electric field induces an electrostatic force capable of overcoming the capillary forces at the narrow (500 nm) aperture on the ink meniscus, which then deforms into a droplet. Once the sheer stress overcomes the surface tension, the droplet then can turn into a Taylor cone^17,27^. This external electric field can be tuned to obtain different modes of EHD printing. The mode of printing known as cone-jet printing is the most desirable mode for the printing of patterns, as it ensures constant, controlled direct writing of the ink onto a substrate^21^. EHD printing should also be capable of depositing inks of dissolved monolayer compounds for the self-assembly of molecular monolayers, which would allow the integration of this additive manufacturing process with the bottom-up assembly of nanoparticles we have already explored, as illustrated in Figure 4.

**Figure 5:**
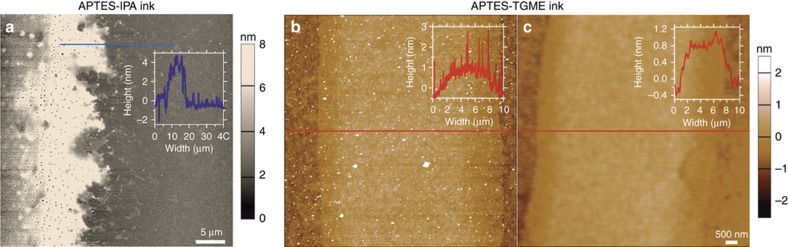
Atomic force microscopy (AFM) measurements of electrohydrodynamic (EHD) printed (3-aminopropyl)triethoxysilane (APTES) films. Topography of APTES films EHD printed from solvents of (**a**) IPA and (**b** and **c**) TGME. (**a** and **b**) were captured using AFM tapping mode and (**c**) was captured in contact mode. Inset lines profiles show the topography for the blue line in **a** and red lines in **b** and **c**. The APTES features in **a** appear to have crystallized and formed relatively large agglomerates 5 nm in size, compared to the 1.2 nm layer observed under both (**b**) tapping and (**c**) contact-mode imaging. Features printed with the TGME ink were also more stable, with irregular lines present for APTES-IPA ink compared to the straighter lines produced from the APTES-TGME ink. This demonstrates that an APTES coating was more consistent and more closely approached that of a monolayer for the TGME ink than for the IPA ink.

The inks for printing were prepared by diluting APTES, the compound for functionalization of the surface, with desired solvents by sonication for 15 min. With ethanol, propan-2-ol (IPA) or aqueous solvents, our results were mixed; jetting of the ink was not achievable with more hydrophilic ethanol and aqueous inks. With an IPA solvent, the results most closely corresponding to a monolayer were achieved with a solution printed from a 5 mM solution of APTES, at a 5 kV bias, a scanning speed of 0.5 mm s^−1^ and a capillary-surface distance of 100 μm. Once printed onto the surface, the surface was conditioned for 12 h in a humid environment to allow the monolayer to grow more effectively across the surface, and then cleaned by sonication in IPA and a subsequent sonication in deionised water. An AFM scan of the results of this is presented in Figure 5a, showing that this formed a very rough, porous surface of APTES with a thickness of up to 5.6 nm at the boundaries (see inset).

**Figure 6:**
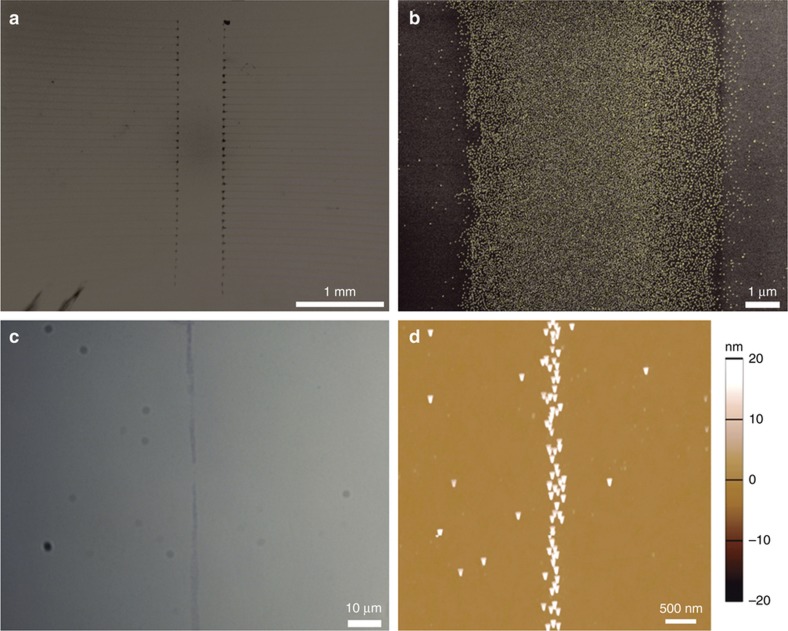
Au Nanoparticles assembled on electrohydrodynamic (EHD) printed (3-aminopropyl)triethoxysilane (APTES). (**a** and **b**) Scanning electron microscopic (SEM) images of 20 nm Au particles assembled onto EHD printed APTES arrays of monolayers. (**a**) Two large arrays of Au nanoparticles on APTES deposited during a raster scan with 75 μm pitch between each consecutive line. (**b**) A close-up image of one of the printed lines in **a**, showing nanoparticles assembled densely onto a 7 μm wide band of APTES. (**c**) An optical microscope image of nanoparticles assembled onto a band of APTES that was printed with a velocity gradient, with the narrowest region formed at the peak (3.8 mm s^−1^) at the centre. (**d**) An AFM topography image of the nanoparticles assembled onto the narrowest section in **c**, with nanoparticles assembled onto an ~300 nm wide band of APTES on the silica surface.

The most effective solvent for EHD printing of APTES utilized in our experiments was triethylene glycol monomethyl ether (TGME). In the presented results, a solution of 50 mM APTES in TGME was printed at a 7 kV bias, a scanning speed of 2 mm s^−1^ and a capillary-surface distance of 30 μm. 15 min after printing the surface was rinsed by sonication in IPA and a subsequent sonication in deionised water. AFM non-contact and contact topography measurements of this print are presented in Figures 5b and c, respectively. These measured the bands of printed APTES to be approximately 8 μm in width and line profile measurements (inset, red lines) measured the printed film thickness at 1.2 nm, corresponding well to that of a molecular monolayer of APTES. These monolayers were quite stable across large scales on the surface (Figure 6a). Some additional, sub-100 nm bands of APTES were observed running parallel to these lines, and were probably the result of multiple jets forming from the nozzle head^28^. The resolution also shows some hysteresis in size, due to the need to maintain a stable electric field, which would require a closed feedback loop and better control of charge build up in the printed ink^29^.

### Printed monolayers for nanoparticle self-assembly

The functionality of these printed APTES surfaces from a TGME-APTES ink was tested to see if electrostatic self-assembly of gold nanoparticles would still occur. After 8 min of assembly of 20 nm Au particles from a droplet deposited on the surface, the surfaces were imaged using SEM and AFM, the results of which are presented in Figure 6. Raster scan printing of APTES, approximately 7 μm wide with 75 μm pitch, are observed to have printed onto the silica surface with SEM following the assembly of nanoparticles (Figure 6a). Printing at these scales appears to be highly consistent, though a feedback is required between printing voltage and the speed of the stage to prevent excess printing during acceleration/deceleration of the stage. A zoomed in image of this surface shows that the Au nanoparticles have clearly assembled densely onto the printed APTES surface and are largely repelled from the uncoated silica surface. The nanoparticles that are present on the repulsive surface may have resulted from random instability or a smaller degree of spreading of APTES in the gas phase as may have been the case with the IPA-APTES ink^30^. Such process variations are to be expected and further investigated to develop fully characterized nanomanufacturing processes.

To achieve a higher resolution, a velocity profile test was implemented at a constant tip-surface voltage of 7 kV. Once a jet had been established, the printbed was moved with respect to the EHD-nozzle over a 75 μm distance which, based on the acceleration profile of the DC motor stage, would have achieved an upper velocity of 3.8 mm s^−1^. One such printed profile is observed optically in Figure 6c, after nanoparticle assembly has occurred, with the narrowest section present at the centre of the printed line. At this speed, the EHD has produced a finer jet onto the surface; closer investigation with AFM (Figure 6d) confirms a width of ~300 nm. Although the stability of this printing resolution could not be maintained across a larger scale, it is clear from these results that EHD printing is capable of writing templates of functional APTES with nanoscale resolution. More stable printing solutions are likely to be achieved through controlled printing of droplets using pulsed deposition^29^.

### DHKPM metrology of monolayers in liquid (in-process nanometrology)

**Figure 7:**
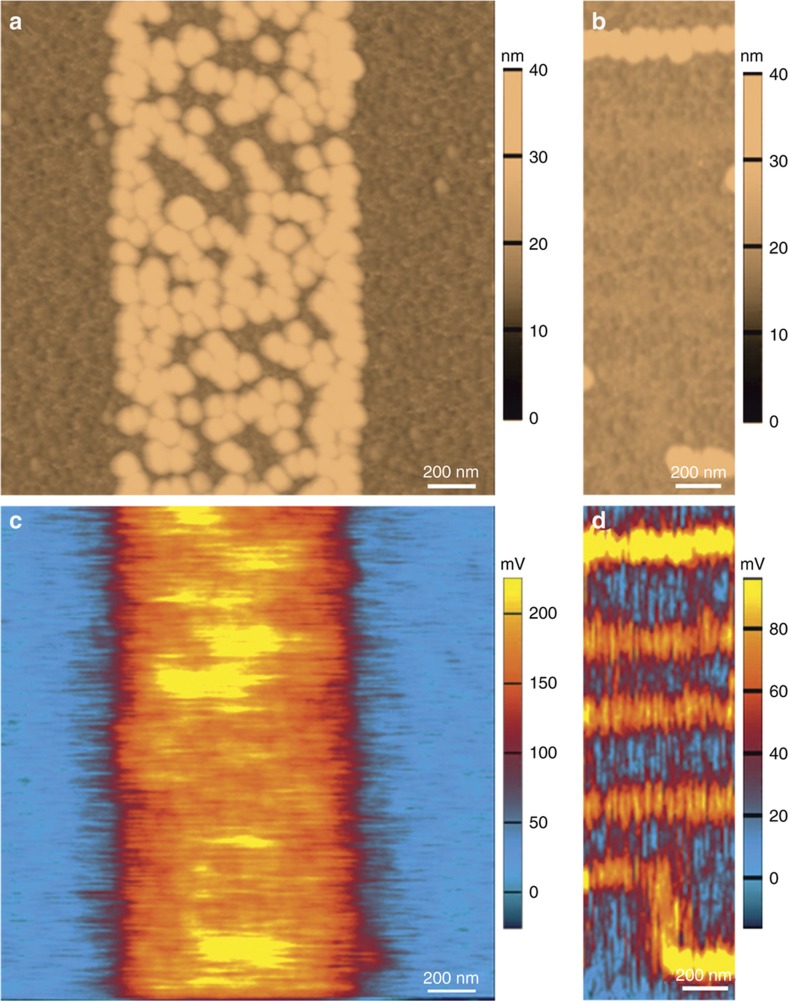
KPM identification of monolayer metrology. (**a** and **b**) Atomic force microscopy (AFM) topography and (**c** and **d**) corresponding Kelvin Probe Microscopy (KPM) surface potential maps of 20 nm Au particles partially assembled from a colloidal droplet (2×10^11^ per mL) over 8 min onto patterned (3-aminopropyl)triethoxysilane (APTES) monolayers. The monolayer is readily identified from the bare SiO_2_ surface and the nanoparticles that cover some of the monolayer, although the surface potential difference measured varied as the morphology of the AFM tip changed.

Once printed, the metrology of the monolayers used in device architectures is an essential aspect of our nanomanufacturing process, particularly to differentiate between different assembled components. However, how does one perform metrology of single monolayers (for example to verify that molecular assembly has occurred, prior to self-assembly)? Conventionally one method of metrology of surface charge is through KPM. In Figure 7, using a two-pass approach to KPM, we have obtained surface potential measurements of an APTES-patterned surface with 20 nm Au particles assembled onto it. This particular assembly was performed over 5 min using a colloid of reduced concentration (1.4×10^11^ per mL, down from 7.0×10^11^ per mL) so the monolayer features are purposefully only partially coated with these particles. In Figure 7a, a 1 μm wide band of APTES down the centre of the image is almost completely coated with particles, but there are gaps to the underlying surface. In the corresponding KPM scan (Figure 7c), we can identify the surface that should have been coated with particles has registered a distinct surface potential from the surrounding silica surface. A similar case is observed in Figure 7b, where 100 nm bands of APTES are patterned on the surface. Some small differences in the topography where the APTES is patterned are visible but are more easily identified in the corresponding KPM image (Figure 7d). It should be noted that between Figure 7c and d, changes to the parameters have changed the apparent difference in surface potential: the monolayer appears to have a higher surface potential than the nanoparticles in Figure 7c and vice versa for Figure 7d.

**Figure 8:**
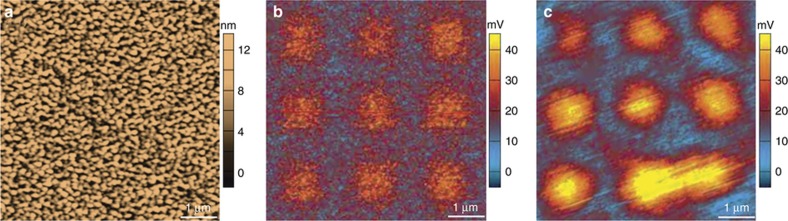
Kelvin Probe Microscopy (KPM) for challenging monolayer metrology. (**a**) Atomic force microscopy (AFM) topography and (**b**) corresponding KPM surface potential map of 1 μm squares of 16-mercaptohexadecanoic acid patterned onto an Au surface. The monolayer is not visible on the rough Au surface in (**a**) but is easily identified in (**b**). (**c**) 16-Mercaptohexadecanoic acid (MHA) monolayers identified using Dual-Harmonic KPM whilst the substrate was immersed in water, which indicates measurements of monolayers on devices during operation in electrolytes could be characterized if required.

In cases where the topography of the monolayer formation on the surface cannot be identified, the metrology of these devices by KPM becomes particularly important. In Figure 8, we look at an example where a surface coated with Au that is subsequently patterned with 1 μm squares of MHA. In the AFM topography scan (Figure 8a) the surface roughness of the Au is quite high, typical of thermally evaporated surfaces used in device fabrication. However, the corresponding KPM scans of this surface clearly distinguish the squares of MHA with a surface potential higher than the surrounding surface. Using DHKPM in Figure 8c, we again identify the MHA monolayers on the Au surface, now with a higher surface potential compared to the measurements in air (Figure 8b) due to the monolayer ionizing in solution. Recent innovations in KPM indicate that further information can be derived in these studies, such as the potential profile of the electrical double layer^21,31^ or dielectric strength of materials^32,33^. DHKPM has demonstrable functionality in electrolytes, which are a necessary component of many devices including the ionic environments of battery electrolytes^21,34^, indicating that such metrology can be performed *in-situ* on monolayer compounds during device operation.

## Discussion

From these experiments we conclude that electrostatic assembly is readily achieved using traditional lithography techniques, assembling nanoparticles onto surfaces in short time scales. The funnelling of nanoparticles into structures to achieve features with smaller critical dimensions than that of the template were achieved, which is consistent with previous predictions made by modeling of nanoparticle assembly^24^. However, for rapid assembly the colloid used must have a high particle concentration, which reduces this funnelling effect. Particle spacing of the order of nanometres is desirable for many applications, particularly single-electron transport, and this spacing could be reduced through shrinking of the electrical double-layer by increasing the ionic strength of the colloidal medium to a critical level. All of these factors should be considered when templating for nanoparticles to assemble into pre-existing surface features in a manufacturing process.

For higher-resolution features, recessed-templating of the attractive surface improves the resolution of the resulting nanoparticle deposition and, in the case of a removable template of PMMA, can remove the inconvenience of the nanoparticles that adhere to the repulsive regions of the template. The use of removable templates with better-defined edges, rather than the rounded edges typical of PMMA following lithography-development, would also likely improve such funnelling. However, for additive printing of masks onto the surface, the direct printing of an attractive molecule for self-assembly is desirable, as the greater time scales required for the negative-tone printing of a repulsive mask would be less efficient.

The SiO_2_ combined with direct EHD printing of the APTES monolayer is clearly effective at enabling the direct deposition of devices for nanoparticle assembly. Although this results in a planar geometry, that as we have demonstrated will provide less funnelling of nanoparticles into printed features, it does enable the additive manufacture of the features in a single-step process, making it more desirable for development. Microscale EHD printing of APTES was achieved reliably with rapid patterning, though the nanoscale patterning was more difficult to maintain over the larger areas, as EHD modes would revert to different modes of spraying at higher print speeds^35^. Developing a reliable nanoscale resolution printer for manufacturing may rely on the NanoDrip method of EHD, which has shown a limited printing velocity of 1–10 μm s^−1^ for 500 nm metal networks^17^. This would have a hugely detrimental impact on the patterning time, whereas the 3–4 mm s^−1^ demonstrated in this paper compares favorably to the upper speeds of 10 mm s^−1^ utilized in electron-beam lithography^36^. The use of emerging methods of EHD printing, such as combinations with AFM-capillaries for closed-loop nanoscale nozzle-surface separation^37,38^, would greatly improve the resolution we show here for continuous printing. This could be used to directly print far denser networks of nanoparticles, opening a route for the use of additive nanomanufacturing to create single-electron device circuitry^5^, sensors^2^, and nanoplasmonic devices^3^. Nanoparticles can also be positioned through this process to enable the assembly of other components, such as the positioning of optoelectronic components^39^.

All these capabilities could be integrated with existing devices through this additive fabrication scheme. Directly printing onto existing devices to add nanomaterial functionalities would be highly enabling to completely blend electrical, optical and biochemical components. For instance, printing sensors directly onto circuits, machinery or medical devices could greatly enhance their functionality. Further refinement of this manufacturing methodology would be able to achieve the sub-50 nm patterning of functional molecules EHD printing is capable of, but the goal of this work is to show that combined processes work just as well as established lithography, and thus need to be considered a serious alternative.

## COnclusions

We have provided a broad scope of the opportunities currently available to integrate high-resolution assembly of nanoparticles, using EHD to achieve rudimentary additive nanomanufacturing. Crucially we demonstrate two key advances: (1) printing molecular monolayers with EHD; and (2) such printed monolayers can subsequently be used to self-assemble nanoparticles onto a surface. We achieve this through the use of a TGME-APTES ink and EHD printing. The printed monolayers were then able to electrostatically self-assemble Au nanoparticles onto these selectively printed surfaces. This approach to manufacturing has great potential to effectively integrate electrical, optical and biochemical components to produce devices with enhanced functionalities. Further, we also present metrology of these printed surfaces, and show that Dual-Harmonic KPM is a highly effective technique for studying molecular monolayers in device architectures, even in disruptive operating environments such as polar solvents. Thus, while the technological opportunities afforded by nanomaterials’ prototyping techniques need substantial development, the combination and extension of existing additive nanomanufacturing techniques into functional nanomaterials and smaller dimensions provides a very promising pathway towards achieving this.
